# KDM8/c-Myc axis-mediated glucose metabolism reprogramming promotes the progression of ovarian cancer

**DOI:** 10.1038/s41598-026-47344-6

**Published:** 2026-04-27

**Authors:** Chunyan Liu, Qian Xu, Zhuoling Li, Xiaoli Yang, Bibo Mao, Lili Guo, Xin Liu, Wenyuan Liu

**Affiliations:** 1 Laboratory of Medicine, The Affiliated Women and Children’s Hospital of Ningbo University, No.339 Liuting Street, Ningbo City, 315012 Zhejiang Province China; 2 Department of Outpatient and Emergency, The Affiliated Women and Children’s Hospital of Ningbo University, Ningbo, 315012 Zhejiang China; 3 Department of Stomatology, The Affiliated Women and Children’s Hospital of Ningbo University, Ningbo, 315012 China

**Keywords:** KDM8, c-Myc, Ovarian cancer, Glucose metabolism, Invasion, Migration, Cancer, Cell biology, Molecular biology

## Abstract

Both KDM8 and c-Myc have been implicated in regulating tumor glucose metabolism. However, whether there is an interaction between KDM8 and c-Myc, and whether KDM8 function is dependent on c-Myc in ovarian cancer (OC) remains unclear. Paired cancerous and paracancerous tissues from five OC patients were analyzed for KDM8 and c-Myc expression using reverse transcription quantitative polymerase chain reaction and Western blot. Co-Immunoprecipitation assays were conducted to verify their potential interaction. Stable OVCAR3 and SKOV3 cell lines overexpressing KDM8 or c-Myc were established. Functional assays (CCK-8, Transwell, colony formation, wound healing, and flow cytometry) were performed to assess proliferation, migration, invasion, colony formation, and apoptosis. Metabolic changes were evaluated by measuring glucose uptake and lactate accumulation using colorimetric and ELISA kits, respectively. Finally, a nude mouse subcutaneous xenograft model was constructed to observe the growth and metabolic levels of OC in vivo. Both mRNA and protein levels of KDM8 and c-Myc were significantly upregulated in ovarian cancer (OC) tissues compared to paracancerous tissues. Furthermore, a direct interaction between KDM8 and c-Myc was identified. Functionally, KDM8 and c-Myc synergistically promoted the malignant behavior of OC cells, including enhanced proliferation, migration, invasion, and colony formation capacities. Additionally, they promoted metabolic reprogramming, as evidenced by increased glucose uptake and lactate accumulation, while concurrently inhibiting apoptosis. However, siRNA-mediated knockdown of c-Myc significantly attenuated these oncogenic effects, reversing the enhanced proliferative, migratory, and metabolic capacities of OC cells. In vivo experiments further verified that the KDM8 /c-Myc axis affects OC growth and metabolic levels. Collectively, our findings indicate that KDM8 and c-Myc cooperate to promote OC progression, which may be mediated through the regulation of glucose metabolism. Notably, KDM8 function is partially dependent on c-Myc in this context. These results provide preliminary evidence supporting KDM8 as a potential candidate target for OC diagnosis and treatment, with further validation required in larger cohorts.

**Supplementary Information:**

The online version contains supplementary material available at 10.1038/s41598-026-47344-6.

## Introduction

Ovarian cancer (OC) represents one of the most prevalent gynecological malignancies, with an estimated 250,000 new cases diagnosed each year globally. Its incidence ranks third among gynecological cancers, trailing only endometrial and cervical cancers. Despite surgical resection and radiotherapy remaining the cornerstone of standard clinical management for OC to date, the 5-year overall survival rate of affected patients remains dismally low at approximately 30%. OC-associated mortality accounts for the fifth highest rate among all malignant tumors and claims the top position among gynecological malignancies^1,2^. Therefore, it is of great significance to explore the molecular mechanisms of OC occurrence and development, and to search for important biomarkers for early diagnosis, monitoring of treatment effects and prognostic assessment of ovarian cancer in order to improve the current situation of OC.

Alteration of glucose metabolism characterized by increased aerobic glycolysis (also known as Warburg effect) has been well-established as one of the hallmarks of cancer. The accumulation of lactate and enhanced glucose uptake in cancer cells represent key hallmarks that drive cancer development. These two metabolic alterations synergistically regulate energy metabolism, remodel the tumor microenvironment, and modulate malignant biological behaviors, thereby collectively promoting the progression, invasion, and chemoresistance of cancers^3,4^. Glucose metabolism reprogramming has been reported to be implicated in various human cancers, such as pancreatic cancer^5^, colorectal cancer^6^, breast cancer^7^, and OC^8^. Recently, a paradigm shifts in our understanding of OC biology with more and more recognition that metabolic reprogramming is not merely a consequence of malignancy but a fundamental driver of tumor progression, immune evasion, and therapeutic resistance^9,10^. KDM8, a histone lysine demethylase, is highly expressed across multiple cancer types, and has been implicated in the regulation of both cell cycle and tumor metabolism regulation in cancer cells^11–16^. In a recent study, KDM8 is found to promote OC invasion and metastasis by binding to the promoter region of glycolysis-associated hexokinase 2 (HK2) and activating HK2 transcription to regulate aerobic glycolysis^17^. Meanwhile, the gene expression level of HK2 is regulated by c-Myc transcription factor. However, whether KDM8 interacts with c-Myc and whether KDM8 regulation of HK2 expression is dependent on c-Myc remains unclear.

In this study, we aim to further explore the relationship between KDM8 and c-Myc, and to detect the protein levels of KDM8 and c-Myc by clinical samples. The corresponding overexpression plasmids and siRNA were constructed to transfect ovarian cancer cell lines to explore the regulatory effects of KDM8 and c-Myc on OC cell proliferation, invasion, migration and glucose metabolism. Specifically, our work seeks to elucidate whether KDM8 promotes OC invasion and metastasis by regulating c-Myc transcriptional activity, thereby reprogramming cellular glucose metabolism. Collectively, these findings are expected to provide a solid theoretical rationale for the potential utility of KDM8 as a novel diagnostic biomarker and therapeutic target for ovarian cancer.

## Materials and methods

### Clinical sample collection

OC tissues and para-cancerous tissues were collected from 5 patients with definite pathological diagnosis. Informed consent was obtained from all subjects and/or their legal guardian(s). The study was approved by the Ethics Committee of The Affiliated Women and Children’s Hospital of Ningbo University (No.EC2024-042) and complied with the principles outlined in the Declaration of Helsinki.

### Cell culture and transfection

Human OC cell lines (OVCAR3, SKOV3, ES-2, HEY) and normal ovarian epithelial cell line HOSEpiC were obtained from American Type Culture Collection (Manassas, VA, USA) and maintained in RPMI 1640 medium (Thermo Fisher Scientific, Waltham, MA, USA) supplemented with 10% FBS at 37 °C under a humidified 5% CO_2_ atmosphere.

pCDNA3.1-CMV-hKDM8-HA, pCDNA3.1-CMV-c-Myc-Flag, pCDNA3.1-KDM8 (OE-KDM8), pCDNA3.1-c-Myc (OE-c-Myc) and siRNA c-Myc (siRNA c-Myc-1, forward: 5’-CGACGAGACCUUCAUCAAA-3’, reverse: 5’-UUUGAUGAAGGUCUCGUCG-3’; siRNA c-Myc-2, forward: 5’-CCAAGGUAGUUAUCCUUAA-3’, reverse: 5’-UUAAGGAUAACUACCUUGG-3’; siRNA c-Myc-3, forward: 5’-GGAACUAUGACCUCGACUA-3’, reverse: 5’-UAGUCGAGGUCAUAGUUCC-3’) as well as the corresponding negative control were provided by GenePharma (Shanghai, China). Following the manufactures’ instructions, OVCAR3 and/or SKOV3 cells were transfected with the above reagents using Lipofectamine 3000 (Thermo Fisher Scientific).

### Measurement for glucose uptake and lactate levels

Following the manufacturer’s protocol, glucose uptake and lactate levels were assessed using Glucose Assay Kit and Lactate ELISA Kit (Nanjing Jiancheng BIO, China), respectively.

### Seahorse assay

We performed seahorse assays to quantitatively measure extracellular acidification rate (ECAR) and oxygen consumption rate (OCR) of individual groups of cells using Seahorse XF Glycolysis Stress Test Kit (Seahorse Bioscience, Billerica, MA, USA) and Seahorse XF Cell Mito Stress Test Kit (Seahorse Bioscience) in the Seahorse XFe 96 Extracellular Flux Analyzer (Seahorse Bioscience), following the manufacturer’s protocols. Briefly, individual groups of cells (1 × 10^4^ cells/well) were cultured into a Seahorse XF 96-well microplate and their baseline measures were determined. Subsequently, the cells were treated sequentially with glucose, oligomycin (the oxidative phosphorylation inhibitor), and 2-DG (the glycolytic inhibitor) at indicated dose and time points for measurement of ECAR. Similarly, the cells were treated sequentially with oligomycin, oxidative phosphorylation FCCP (carbonyl cyanide-p-trifluoromethoxyphenylhydrazone, the reversible inhibitor), and rotenone/antimycin A (Rote/AA, the mitochondrial complex I inhibitor and the mitochondrial complex III inhibitor, respectively). Data were analyzed by Seahorse XF-96 Wave software and expressed as pmols/min for OCR and mpH/min for ECAR, respectively.

### Cell counting kit-8 (CCK-8)

CCK8 assay was used to assess OC cell proliferation. OC cells (1 × 10^3^/well) were seeded in a 96-well plate, with 3 replicate wells per sample. The plate was incubated at 37 °C with 5% CO_2_ for 24 h to allow cell adhesion. Afterward, the original medium was replaced with fresh medium containing 10% CCK8 reagent (Dojindo, Japan). Then, the 96-well plate was further incubated at 37 °C for 2 h. The absorbance at 450 nm was measured using a microplate reader (Thermo Fisher Scientific).

### Reverse transcription quantitative polymerase chain reaction (RT-qPCR)

Total RNA was extracted from cells using Trizol regent (Invitrogen, Carlsbad, CA, USA) following the manufacturer’s instructions. cDNA synthesis was performed with the PrimeScript RT reagent Kit (Takara, Dalian, China). A RT-qPCR assay was conducted to assess mRNA expression utilizing the SYBR Premix Ex TaqTM II (Takara). All mRNA expression was normalized to the housekeeping gene GAPDH. The 2^-ΔΔCT^ method was used to evaluate the relative expression levels of each sample. The primers were summarized into Table 1.

**Table 1:** The primers of target genes.

Name	Sequence(5’-3’)
GAPDH	CGGGAAGGAAATGAATGGGC
	GGAAAAGCATCACCCGGAGG
KDM8	GCGCGGGTTTTATACTCTGC
	TGCATTTCTCGCGGCTCATA
c-Myc	TTCATAACGCGCTCTCCAAGTA
	AGAGCGTGGGATGTTAGTGT

### Transwell invasion assay

Transwell chambers (Millipore, Billerica, MA, USA) were used to assess the invasion of OC cells. First, the chamber was coated with Matrigel (BD Biosciences, Franklin Lakes, NJ, USA). OC cells (5 × 10^4^) in serum-free medium were seeded to the upper chamber, while the lower compartment was filled with DMEM medium containing 10% FBS. After 24 h of invasion, cells on the lower surface of the chamber were fixed, stained with 0.5% crystal violet, visualized under a light microscope (OLYMPUS, Tokyo, Japan). Cell numbers were quantified by counting cells in four randomly-selected microscope fields.

### Colony formation assay

OC cells (200 cells/well) were seeded in 6-well plates and cultured for 10–14 days (medium replaced timely based on cell morphology and growth status) until cell colonies formed at the plate bottom. Cells were then fixed with 4% paraformaldehyde, and stained using 1% crystal violet. Finally, the numbers of colonies were imaged and counted under a light microscope (OLYMPUS).

### Wound healing assay

The migration of OC cells was evaluated via wound healing assay. Cells (1 × 10^6^ cells/well) were seeded in 6-well plates and incubated at 37 °C for 24 h to form cell monolayers. Wounds were created by scratching the monolayers with a sterile 200 μL pipette tip. The wounded areas were imaged under a light microscope (OLYMPUS) at 0 and 24 h post-scratching.

### Flow cytometer analysis

OC cells were seeded in 6-well plates. Afterward, cells were stained with Annexin V-AbFluor™ 488/propidium iodide (Abbkine, Beijing, China) following the kit instructions, then analyzed for apoptosis via a flow cytometer (BD Biosciences). For cell cycle detection, OC cells in 6-well plates were fixed overnight in pre-cooled 75% alcohol. After fixation, cells were stained using the Cell Cycle Staining Kit (Abbkine) and analyzed by flow cytometer (BD Biosciences).

### Western blot analysis and co-immunoprecipitation (Co-IP) assay

Protein samples (30 μg) were loaded per lane and separated by 10% SDS-PAGE. Separated proteins were transferred to polyvinylidene difluoride membranes (Millipore). Membranes were blocked with 5% non-fat milk for 1 h at room temperature, then incubated overnight at 4 °C with primary antibodies against KDM8 (cat.no. 20999-1-AP; 1:1000; Proteintech, Wuhan, China), c-Myc (cat.no. 13987S; 1:1000; Cell Signaling Technology, Boston, MA, USA), GAPDH (cat.no. 60004-1-Ig; 1:5000; Proteintech). After washing, membranes were incubated for 1 h at room temperature with horseradish peroxidase (HRP)-conjugated secondary antibodies (anti-mouse: cat. no. A21010; anti-rabbit: cat. no. A21020; both 1:10,000; Proteintech). Blots were visualized using a chemiluminescent system (5200Multi, Tanon), and band densities were quantified with ImageJ software (National Institutes of Health, Bethesda, MD, USA). The Co-IP protocol was consistent with Western blot, except that IgG (cat.no. 98136-1-RR; 1:50,000; Proteintech), anti-HA (cat.no. ABT2040; 1:5000; Abbkine) and anti-Flag (cat.no. ABT2010; 1:5000; Abbkine) antibodies were used for immunoprecipitation.

### In vivo nude mouse subcutaneous tumor experiment

A total of 20 female BALB/c nude mice (four-week-old; weighting 13–15 g) were obtained from SLAC Laboratory Animal Co., Ltd. (Shanghai, China). After 1 week of acclimatization, mice were randomly divided into 4 experimental groups (n = 5): control, OE-KDM8, sh-c-Myc, and OE-KDM8 + sh-c-Myc groups. A subcutaneous tumor model was established by injecting SKOV3 cells (5 × 10^5^ cells/per mouse) that transfected with OE-KDM8 (GenePharma; Shanghai, China), sh-c-Myc (5’-CGACGAGACCTTCATCAAATTCTCGAGAATTTGATGAAGGTCGAGTCGTTTTTT-3’; GenePharma), or OE-KDM8 + sh-c-Myc into the dorsal flank. From day 10 post-injection, tumor volume was monitored regularly. A decrease in normal body weight > 20% was defined as a humane endpoint; none of the mice reached the humane endpoint. On day 30, all mice received an intraperitoneal injection of pentobarbital sodium (200 mg/kg) for euthanasia, and tumor tissues were collected for further analysis. This animal experiment was conducted in compliance with the National Institutes of Health Guide for the Care and Use of Laboratory Animals and approved by the Ethics Committee of The Affiliated Women and Children’s Hospital of Ningbo University (No.EC2024-042). This study is reported in accordance with ARRIVE guidelines.

### HE staining

Mouse tumor tissues were fixed, embedded, and sectioned. Sections were firstly deparaffinized twice with xylene (20 min each, room temperature), then hydrated in a gradient of 100% → 100% → 95% → 90% → 80% → 70% ethanol (5 min each) followed by distilled water for 5 min. They were stained with hematoxylin for 5 min, rinsed with alkaline PBS for 2 min, then with running water for 3 min. Next, sections were stained with eosin for 10 min at room temperature, quickly rinsed with distilled water, and dehydrated via quick rinses in 70%, 80% and 90% ethanol, followed by 95% ethanol for 30 s and two washes in 100% ethanol for 3 min. After two xylene washes (5 min each), sections were mounted with a neutral mounting medium, photographed under a microscope (OLYMPUS), and documented.

### Statistical analysis

In vitro experiments were performed in triplicate, and each experiment was repeated three times. In vivo experiments were performed using five mice per group. All the data were statistically analyzed using GraphPad Prism software (Graph Pad Software, Inc., USA). The measurement data are expressed as the means ± standard deviations. Comparisons between two groups were made using Student’s t-test, and multiple comparisons were made using one-way ANOVA, followed by Tukey’s post hoc test. The results were considered statistically significant at a* P* value < 0.05.

## Results

### KDM8 and c-Myc are overexpressed in OC tissues and cell lines

**Fig. 1:**
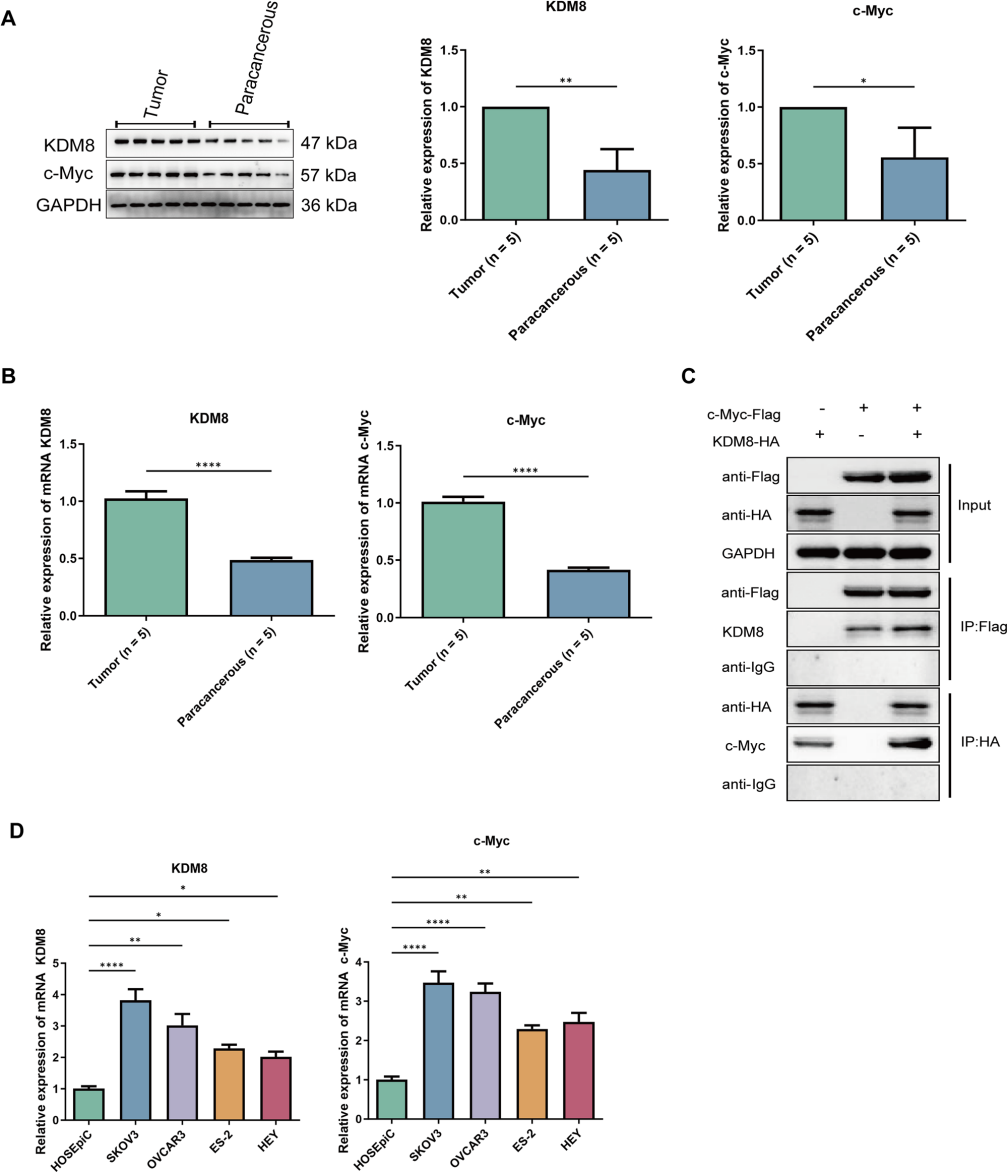
KDM8 and c-Myc are overexpressed in OC tissues and cell lines. (**A**) The protein levels of KDM8 and c-Myc in tumor and paracancerous tissues were measured by western blot. (**B**) The mRNA expression of KDM8 and c-Myc in tumor and paracancerous tissues was detected by RT-qPCR. (**C**) CO-IP assay was used to detect the interaction between KDM8 and c-Myc proteins. (**D**) The mRNA expression of KDM8 and c-Myc in OC cell line was detected by RT-qPCR. ^*^P < 0.05, ^**^P < 0.01, ^****^P < 0.0001.

We firstly collected cancerous and paracancerous tissues from five OC patients. The expression of KDM8 and c-Myc in OC tissues was significantly higher than that in paracancerous tissues, both at the protein level (Fig. 1A) and the mRNA level (Fig. 1B) and. Meanwhile, we transiently co-transfected pCDNA3.1-CMV-hKDM8-HA and pCDNA3.1-CMV-c-Myc-Flag overexpression plasmids into 293 T cells via liposomes, and verified the interactions between KDM8 and c-Myc by using Co-IP technology (Fig. 1C). Additionally, four human OC cell lines (OVCAR3, SKOV3, ES-2, HEY) were included in this study to further determine KDM8 and c-Myc expression levels. As illustrated in Fig. 1D, we found that compared to HOSEpiC, KDM8 and c-Myc expression levels were significantly increased in these four cell lines. We selected OVCAR3 and SKOV3 cell lines for the succeeding functional experiments in vitro due to the relatively high expression levels of KDM8 and c-Myc in these two OC cell lines. These results suggested that KDM8 and c-Myc may co-regulate the progression of OC.

### KDM8 interacts with c-Myc in OC cells

**Fig. 2:**
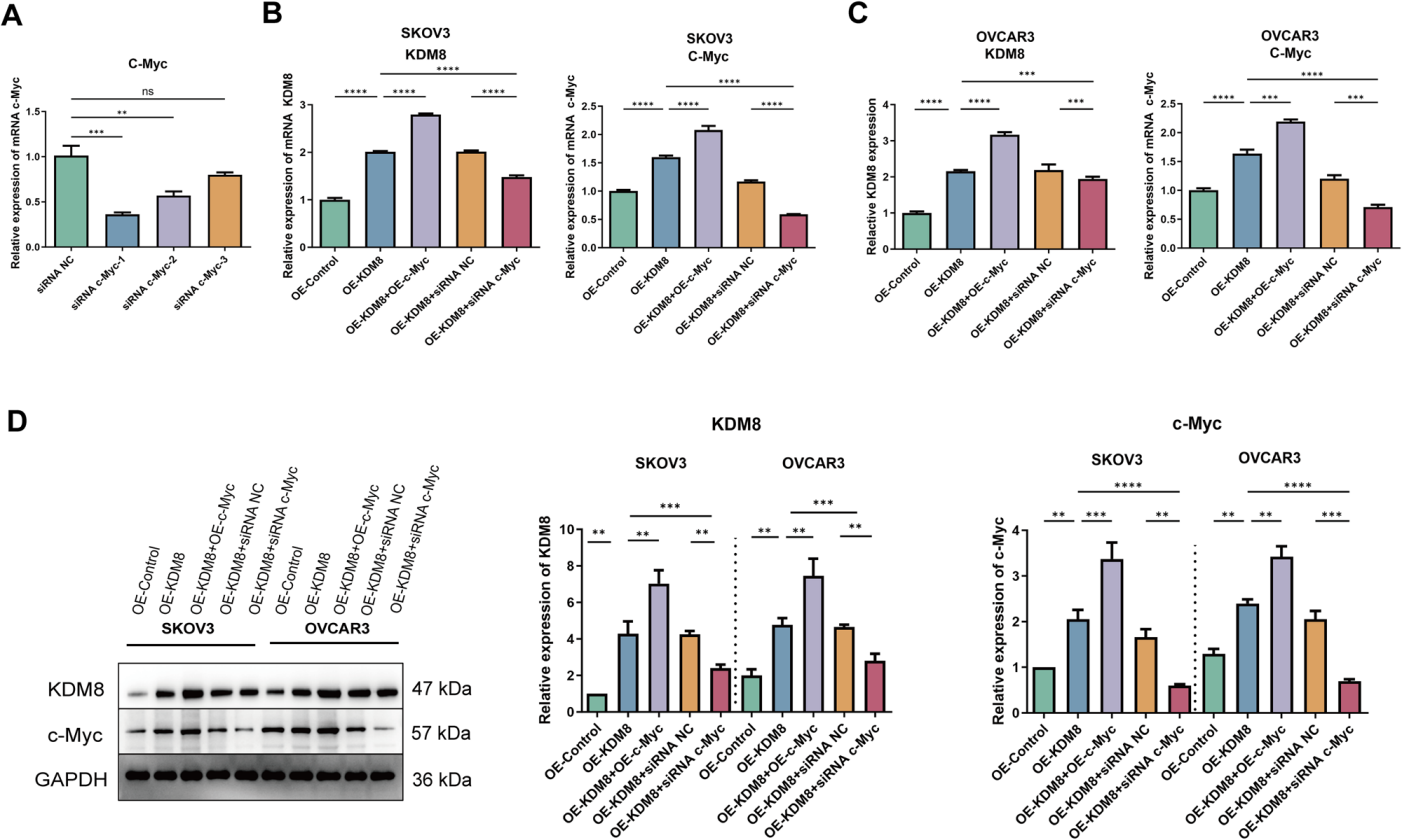
KDM8 interacts with c-Myc in OC cells. (**A**) The knockdown efficiency of siRNA c-Myc was determined via RT-qPCR. Following transfection of OE-KDM8, OE-c-Myc, or siRNA c-Myc, the mRNA expression of KDM8 and c-Myc in (**B**) SKOV3 and (**C**) OVCAR3 cells was detected by RT-qPCR. (**D**) The protein levels of KDM8 and c-Myc in OC cells were measured by western blot. ^**^P < 0.01, ^***^P < 0.001, ^****^P < 0.0001. ns: no significance.

Based on the above experiments that KDM8 underwent protein interactions with c-Myc, OE-KDM8, OE-c-Myc and siRNA c-Myc were transfected into OVCAR3 and SKOV3 cells, and the interaction between KDM8 and c-Myc was further explored. Firstly, the knockdown efficiency of siRNA c-Myc was determined via RT-qPCR. We selected siRNA c-Myc-1 for subsequent experiments due to its relatively high knockdown efficiency (Fig. 2A). As shown in Fig. 2B-C, KDM8 overexpression upregulated c-Myc mRNA expression in both OVCAR3 and SKOV3 cells, while c-Myc overexpression further promoted KDM8 expression. However, transfection with siRNA c-Myc significantly reversed the promoting effect of KDM8 overexpression on KDM8 expression. Protein expression levels demonstrated the same results (Fig. 2D).

### KDM8 interacts with c-Myc to mediate abnormal glucose metabolism, proliferation, apoptosis, invasion, and migration in OC cells

**Fig. 3:**
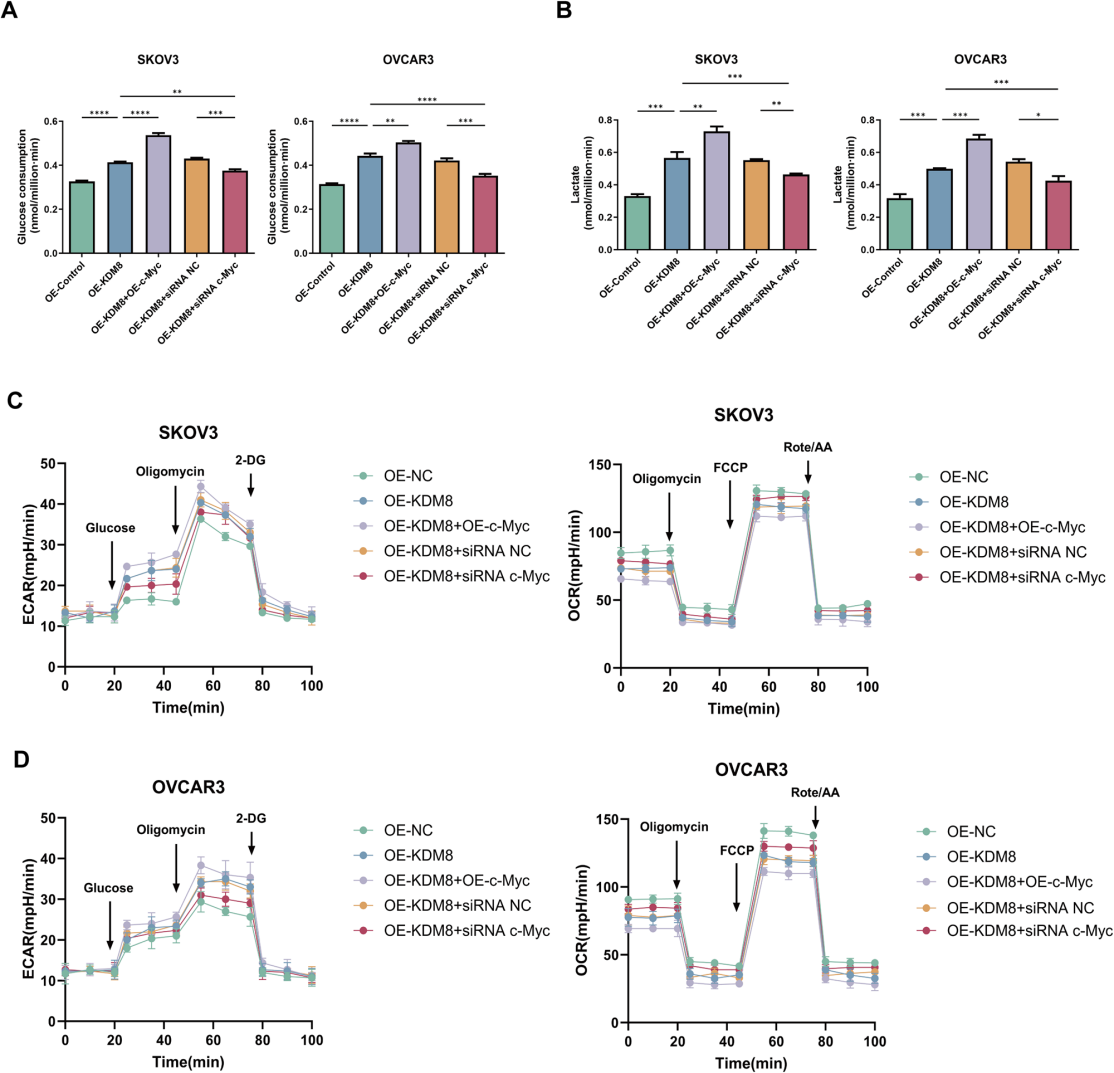
Effects of KDM8 or c-Myc on glucose metabolism of OC cells. (**A**) Glucose uptake assay in OVCAR3 and SKOV3 cells. (**B**) Lactate accumulation assay in OVCAR3 and SKOV3 cells. (**C**-**D**) ECAR and OCR in OC cells were assessed by seahorse assays. ^*^P < 0.05, ^**^P < 0.01, ^***^P < 0.001, ^****^P < 0.0001.

**Fig. 4:**
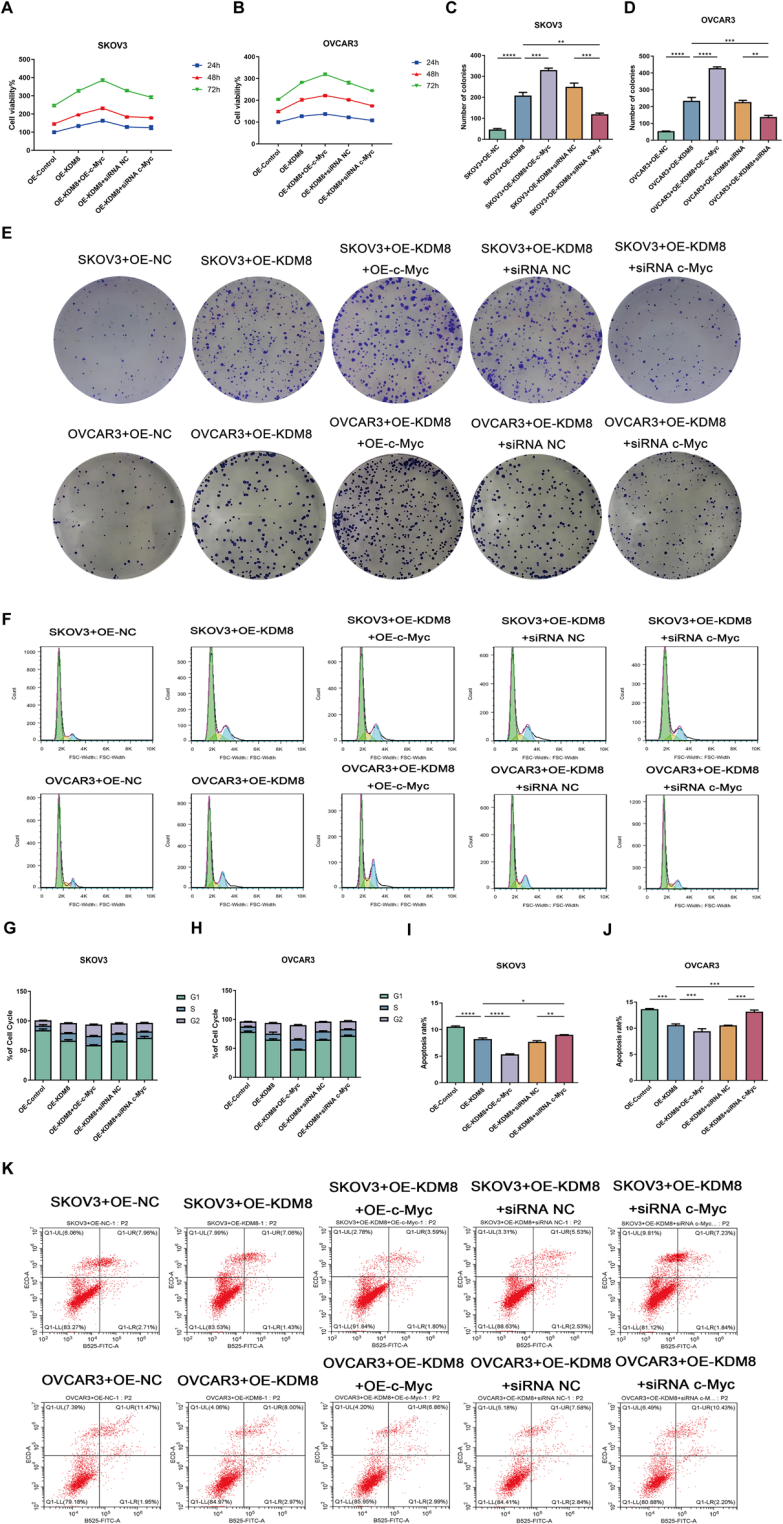
Effects of KDM8 or c-Myc on the proliferation and apoptosis of OC cells. The proliferation of OC cells was measured by (**A**-**B**) CCK-8 assay, (**C**-**E**) colony formation assay, and (**F**–**H**) cell cycle assay, respectively. (**I**-**K**) The apoptosis of OC cells was analyzed by Flow cytometry. ^*^P < 0.05, ^**^P < 0.01, ^***^P < 0.001, ^****^P < 0.0001.

**Fig. 5:**
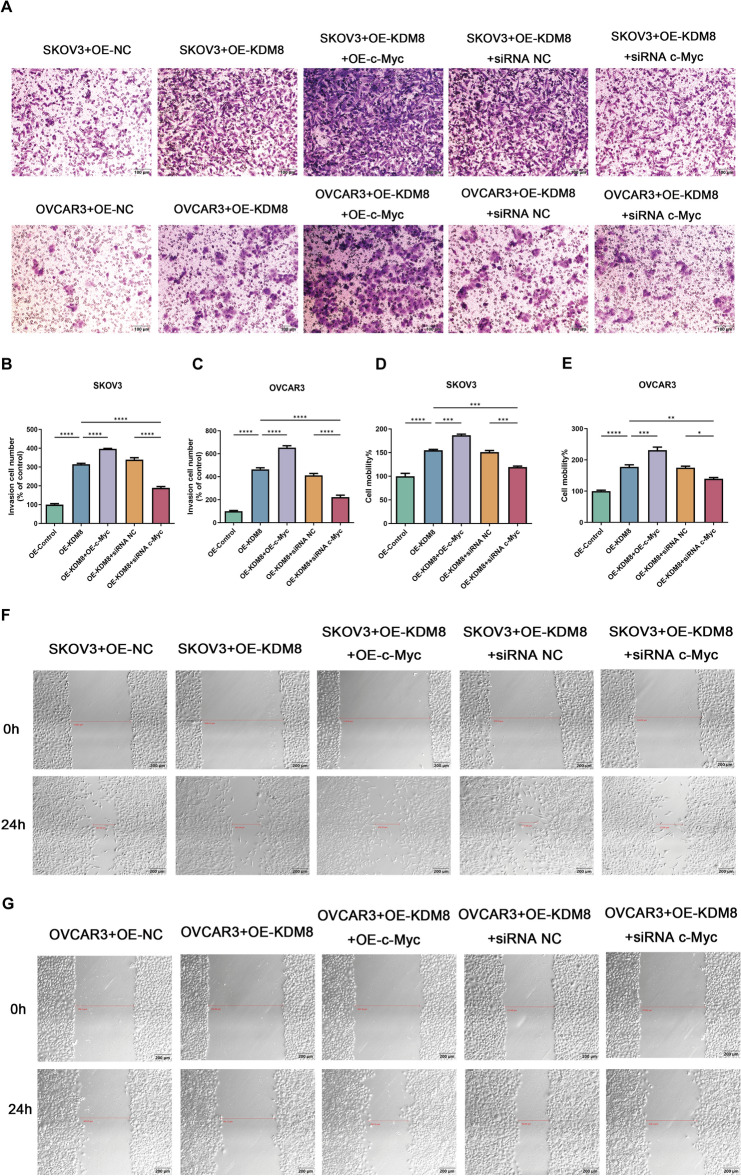
Effects of KDM8 or c-Myc on the invasion and migration of OC cells. (**A**-**C**) The invasive capacities of OC cells were assessed by transwell invasion assay. Scale bar = 100 μm. (**D**-**G**) The migratory capacities of OC cells were assessed by wound healing assay. Scale bar = 200 μm.^*^P < 0.05, ^**^P < 0.01, ^***^P < 0.001, ^****^P < 0.0001.

The functions of KDM8 and c-Myc in OC cells were then assessed. Firstly, we examined the glucose uptake and lactate efflux in OVCAR3 and SKOV3 cells. As illustrated in Fig. 3A-B, overexpression of KDM8 or c-Myc synergistically stimulated the glucose consumption and lactate efflux in OVCAR3 and SKOV3 cells. Meanwhile, silencing of c-Myc was observed to partially counteract the effect of KDM8 overexpression on glucose consumption and lactate efflux. Seahorse assay was performed to further assess the effects of KDM8/c-Myc on glucose metabolism. As illustrated in Fig. 3C-D, compared to the OE-NC group, OE-KDM8 significantly elevated ECAR, but reduced OCR. Co-overexpression of KDM8 and c-Myc further increased ECAR and decreased OCR, suggesting a synergistic promotion of glycolysis by KDM8 and c-Myc. Conversely, c-Myc silencing notably reversed the effects of KDM8 overexpression on ECAR and OCR. Furthermore, the overexpression of KDM8 and c-Myc was found to be synergistically promote the proliferation (Fig. 4A–H) and inhibit apoptosis (Fig. 4I–K), as well as enhance invasion (Fig. 5A–C) and migration (Fig. 5D–F). In contrast, these regulatory effects were remarkably reversed following transfection of siRNA c-Myc.

### Targeting the KDM8/c-Myc axis affects tumor growth in mouse in vivo

**Fig. 6:**
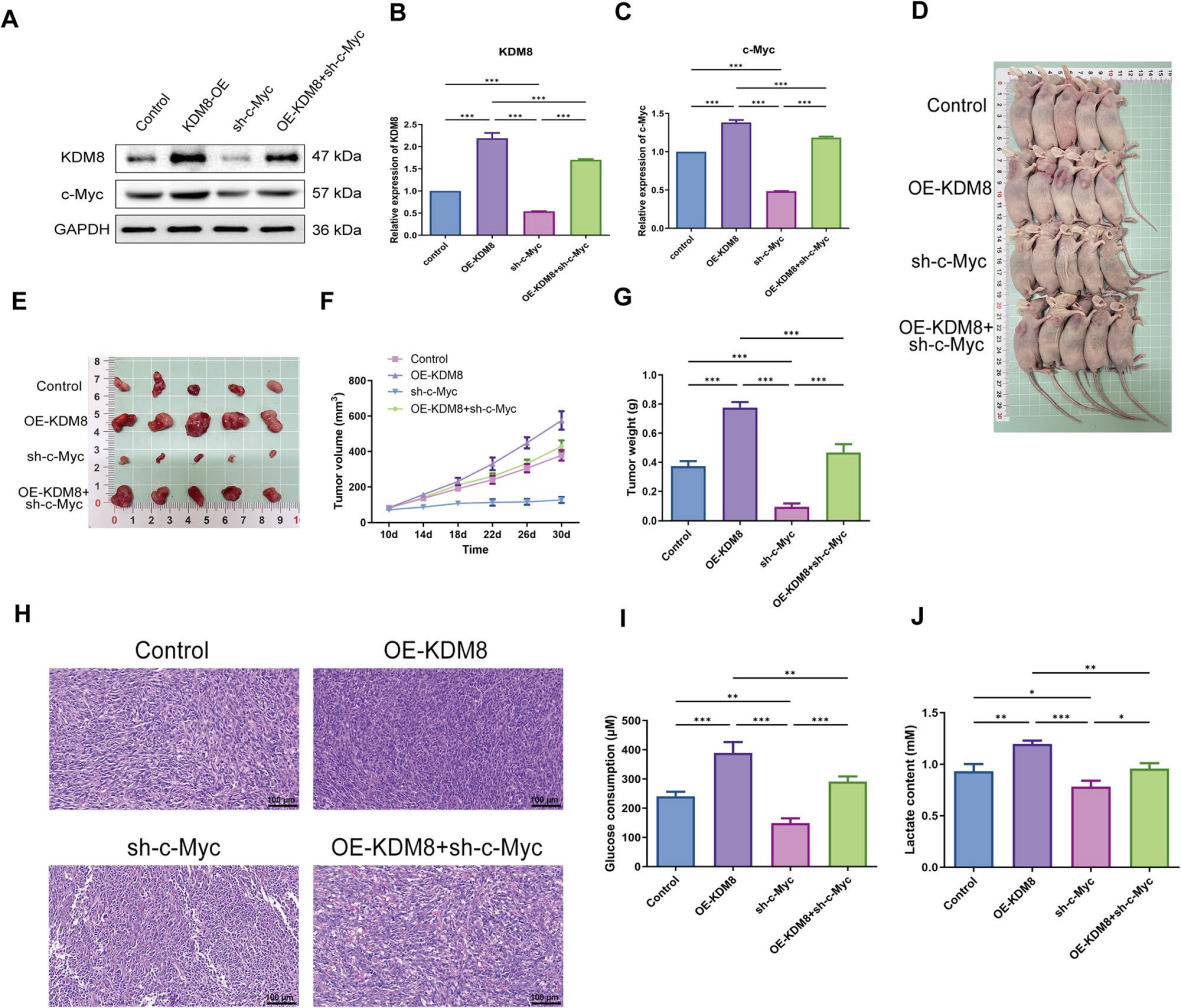
Targeting the KDM8/c-Myc axis affects tumor growth in mouse in vivo. Following injection of OE-KDM8 or sh-c-Myc, (**A**–**C**) The protein levels of KDM8 and c-Myc in xenografts were measured by western blot. (**D**-**E**) The representative images of nude mice and xenografts. (**F**-**G**) Tumor volume and weight were measured. (**H**) HE staining was used to assess tumor histopathology. Scale bar = 100 μm. (**I**-**J**) Glucose consumption and lactate content in xenografts were measured by the corresponding commercial Kits.^*^P < 0.05, ^**^P < 0.01, ^***^P < 0.001.

To further validate the effect of targeting KDM8/c-Myc axis on OC progression in vivo, we constructed a subcutaneous tumor model using nude mice. The protein levels of KDM8 and c-Myc were markedly elevated following injection of OE-KDM8, but were significantly reduced when sh-c-Myc was injected. Meanwhile, silencing of c-Myc in mice remarkably reversed the promoting effects of KDM8 overexpression on KDM8 and c-Myc protein levels (Fig. 6** A-C**). As demonstrated in Fig. 6D-G, tumor progression was significantly accelerated in overexpressing KDM8 mice compared to the controls, while knockdown of c-Myc showed the opposite result. Meanwhile, knockdown of c-Myc to some extent reversed KDM8-mediated tumor growth. In addition, as illustrated in Fig. 6H, we further assessed tumor histopathology by HE staining. In the control group, a small number of tumor cells were necrotic, with different sizes and morphologies, uneven distribution, and a small amount of inflammatory cell infiltration in the mesenchyme. In the OE-KDM8 group, the tumor cells grew vigorously, with irregular nuclear shapes and increased chromatin. Inflammatory cell infiltration was obviously in the mesenchyme. The tumor cells in the sh-c-Myc group exhibited a sparse distribution, with a significant number undergoing necrosis. Interestingly, compared with the OE-KDM8 group, tumor cells in the OE-KDM8 + sh-c-Myc group were loosely arranged and displayed evident signs of necrosis, characterized by varying sizes and morphologies. Additionally, a limited infiltration of inflammatory cells was observed. To further investigate the impact of KDM8/c-Myc axis on glucose metabolism, we assessed lactate production and glucose uptake in tumor tissues. Consistent with our in vitro findings, the results demonstrated that KDM8/c-Myc signaling plays a critical role in modulating glycolytic metabolism, as evidenced by significant changes in both lactate accumulation and glucose utilization (Fig. 6I-J).

## Discussion

Metabolic reprogramming ranks among the newly recognized “hallmarks of cancer”. It exerts a pivotal role in driving multiple hallmark malignant biological behaviors of tumor cells, such as proliferation, migration and invasion. Among the diverse patterns of metabolic dysregulation, the most extensively characterized and prevalent subtype is abnormal glucose metabolism^18–21^. Glucose is the main source of energy in the body, and when glucose molecules are transported to the cell, they will ultimately produce pyruvate and adenosine triphosphate (ATP) through glycolysis^22^. In normal cells, pyruvate produced by glycolysis is transported into the mitochondrial matrix, where it is catalyzed by the pyruvate dehydrogenase complex to form acetyl-coenzyme A. This metabolite then enters the tricarboxylic acid cycle, coupling with oxidative phosphorylation to produce ATP^23,24^. In contrast, tumor cells predominantly depend on glycolysis to meet their bioenergetic and biosynthetic demands, and this metabolic pathway is converted in a way that is mainly known as the Warburg effect^25^. Even in an oxygen-rich state, tumor cells rely mainly on anaerobic glycolysis^26^.

Histone demethylases (HDMs) are enzymes capable of removing methylation modifications on histones, and there are primarily categorized into two evolutionarily conserved families: the Lysine-specific demethylase (LSD) and the JmjC domain-containing family (JMJD)^27–29^. The LSD family specifically removes mono- and bis-methylation modifications of histones H3K4 and H3K9, whereas the JMJD family is predominantly responsible for catalyzing the demethylation of tri-methylated lysine residues^30^. KDM8 (also known as JMJD5) is a member of the JMJD family. Mounting evidence from recent investigations has shown that KDM8 plays an important role in tumorigenesis and malignant progression, with well-documented capacities to potentiate tumor cell invasion and metastatic dissemination. For example, KDM8 regulates cell cycle factor CDKN1A to promote hepatocellular carcinoma proliferation^31^. Hsieh et al.^13^ found that targeting the KDM8/CCNA1 axis holds considerable translational potential as a therapeutic strategy for oral squamous cell carcinoma. Beyond its role in cell cycle control and metastasis, KDM8 has emerged as a key regulator of glucose metabolic reprogramming in cancer. Li et al.^32^ reported a positive correlation between KDM8 expression levels and lactate metabolism in hepatocellular carcinoma. Chen et al. also found KDM8 regulates the mutation of PKM2, a key enzyme in glucose metabolism to promote malignant progression of breast cancer^33^. These studies further illustrate that KMD8 is an important molecule and potential therapeutic target for tumor progression.

As a transcription factor, c-Myc is well recognized for its established roles in the regulation of malignant biological behavior and glucose metabolism in tumor cells^34^. Specifically, c-Myc exerts its oncogenic functions by directly or indirectly regulating the transcription of a diverse array of downstream target genes, with its activity tightly modulated by multiple epigenetic modifications including phosphorylation and methylation^35–37^. Accumulating evidence has further delineated the critical involvement of c-Myc in metabolic reprogramming and tumor progression across multiple cancer types. For instance, METTL5 has been demonstrated to stabilize c-Myc by facilitating USP5 translation, thereby driving glucose metabolic reprogramming and accelerating hepatocellular carcinoma progression^38^. NOP2-mediated m5C modification of c-Myc in an EIF3A-dependent manner, which in turn elicits glucose metabolic reprogramming and promotes hepatocellular carcinoma development^39^. In osteosarcoma, P2RX7 enhances c-Myc stabilization to facilitate both tumor progression and glucose metabolic reprogramming^40^. Given the well-documented roles of KDM8 and c-Myc in regulating glucose metabolism, the present study aimed to further dissect the functional relationship between these two molecules. Therefore, we examined the expression of KDM8 and c-Myc in clinical OC specimens. Subsequently, we constructed recombinant plasmids to verify the existence of an interaction between KDM8 and c-Myc. Furthermore, our in vitro and in vivo findings demonstrated that KDM8 and c-Myc synergistically promoted the proliferation, migration, invasion and colony formation ability of OC cells. Importantly, the oncogenic effects of KDM8 were partially dependent on the transcription activity of c-Myc. These observations are consistent with the findings of Fuhrmann et al.^41^, who reported that KDM8 and c-Myc interact physically and function cooperatively to co-regulate the malignant biological behaviors of tumor cells.

This study has several notable limitations that should be acknowledged. First, this research enrolled only 5 patients per group. The small sample size may limit the generalizability of our clinical findings, and larger cohorts are needed in future studies to validate these results. Second, rescue experiments (e.g., c-Myc overexpression in KDM8-knockdown OC cells) would further strengthen the causal relationship by verifying whether restoring c-Myc expression can reverse the phenotypic changes induced by KDM8 depletion. Meanwhile, bidirectional knockdown experiments (i.e., knock down either KDM8 or c-Myc and examine the mRNA and protein expression levels of the other in OC cells) may provide a more comprehensive elucidation of the reciprocal regulatory expression patterns between KDM8 and c-Myc. Third, our study identified that KDM8 and c-Myc can regulate glucose metabolism. Further exploration of downstream molecular cascades (e.g., specific glycolytic enzymes regulated by the KDM8/c-Myc axis) would enhance mechanistic depth. Additionally, the therapeutic potential of targeting the KDM8/c-Myc axis—including its impact on OC patient survival—has not been explored and warrants future preclinical studies.

## Conclusions

In summary, our study demonstrates that KDM8 and c-Myc are concurrently overexpressed and interact in OC tissues. Functional assays indicate that KDM8 and c-Myc cooperate to enhance OC cell proliferation, migration, invasion, and colony formation, while suppressing apoptosis, and these effects are associated with the regulation of glucose metabolism. Importantly, the oncogenic functions of KDM8 in OC are partially dependent on c-Myc. While these findings provide a preliminary theoretical basis for considering KDM8 as a potential candidate target for OC diagnosis and treatment, further validation in larger clinical cohorts, mechanistic studies to elucidate downstream signaling pathways, and preclinical therapeutic trials are essential to confirm the clinical relevance and translational potential of these observations.

## Supplementary Information

Supplementary Information.

## Data Availability

The datasets supporting the conclusions of this article are included within the article (Due to the principle of confidentiality of the article, all data are reasonably accessible to corresponding authors).
