# Supplementary material for: KDM8/c-Myc axis-mediated glucose metabolism reprogramming promotes the progression of ovarian cancer
Source: Sci Rep. 2026 Apr 27;16:15865. doi: 10.1038/s41598-026-47344-6 (PMC13194864; doi:10.1038/s41598-026-47344-6)

Figure 1A

Grouping from left to right: Tumor, Tumor, Tumor, Tumor, Tumor; Paracancerous, Paracancerous, Paracancerous, Paracancerous, Paracancerous

KDM8

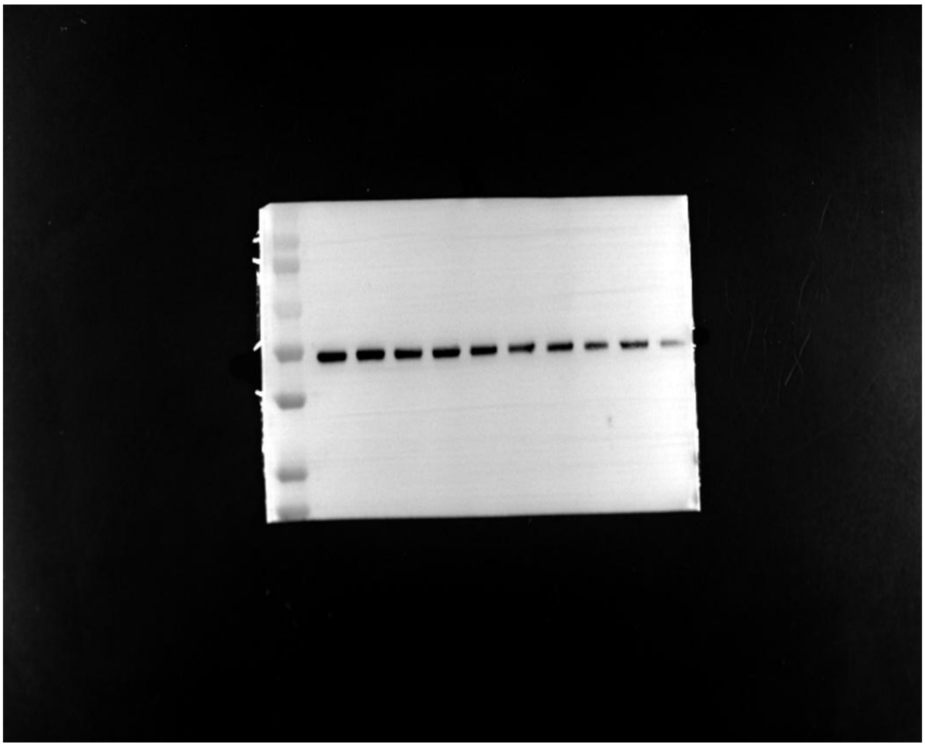

C-Myc

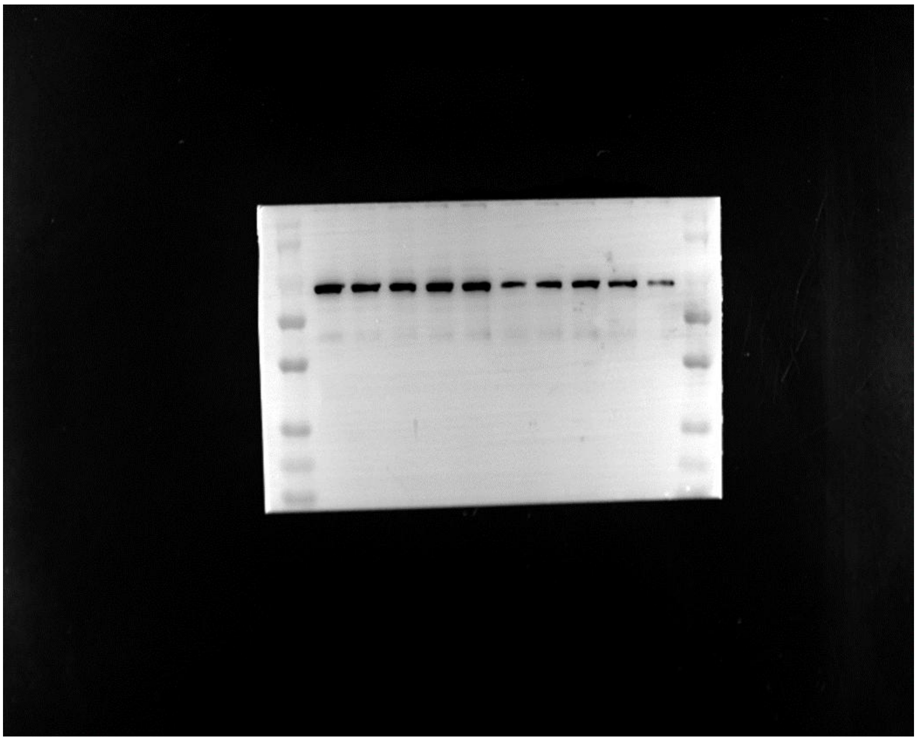

GAPDH

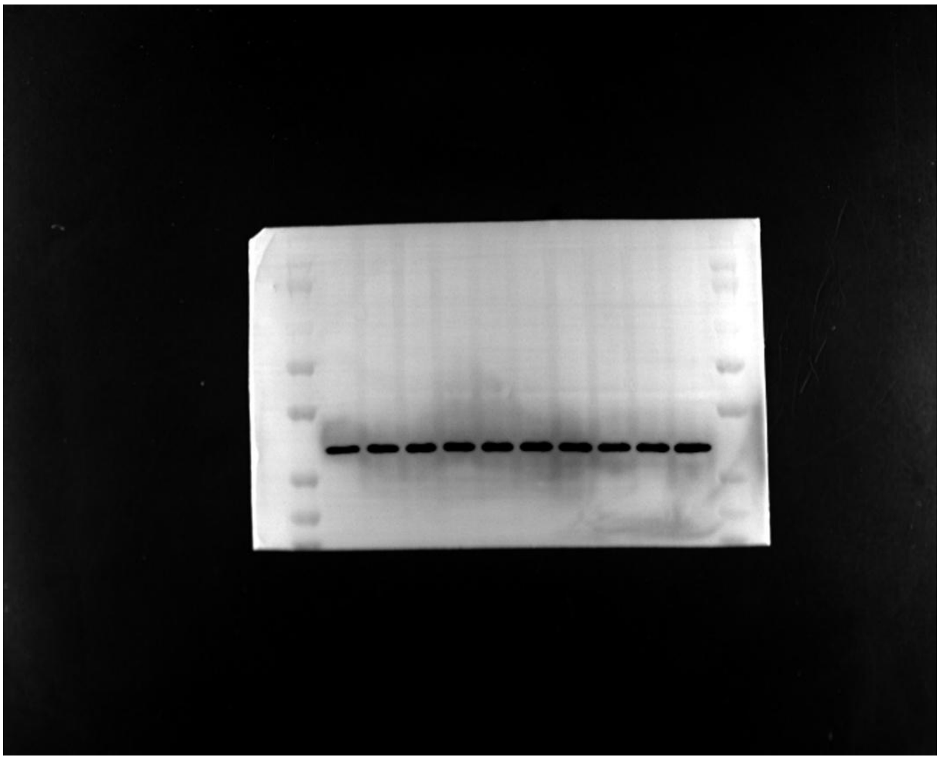

Figure 1C

Input:

anti-Flag

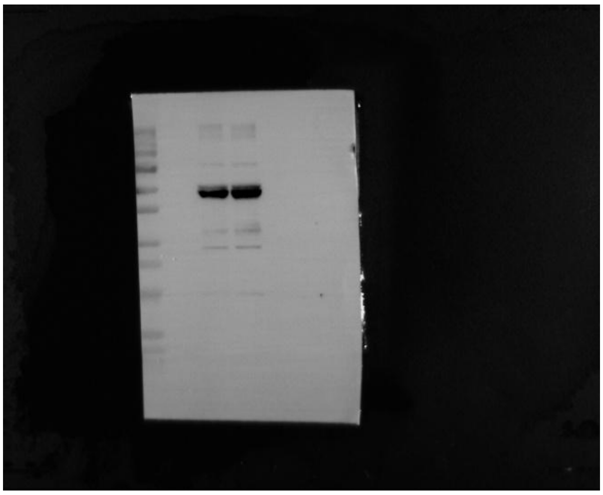

anti-HA

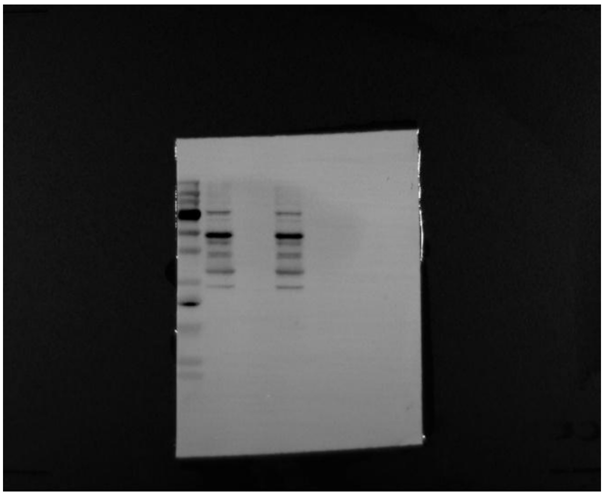

GAPDH

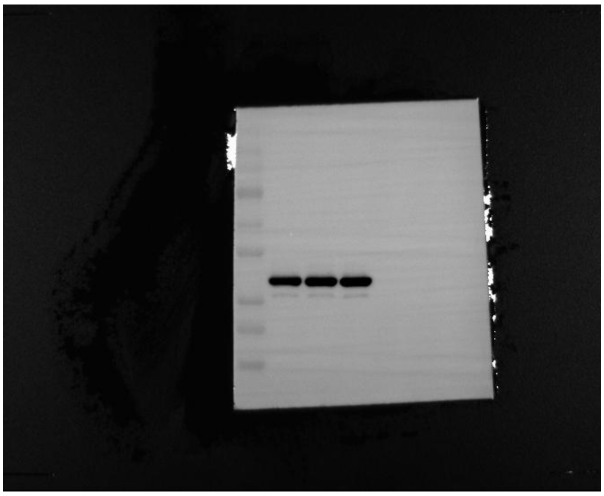

IP: Flag

anti-Flag

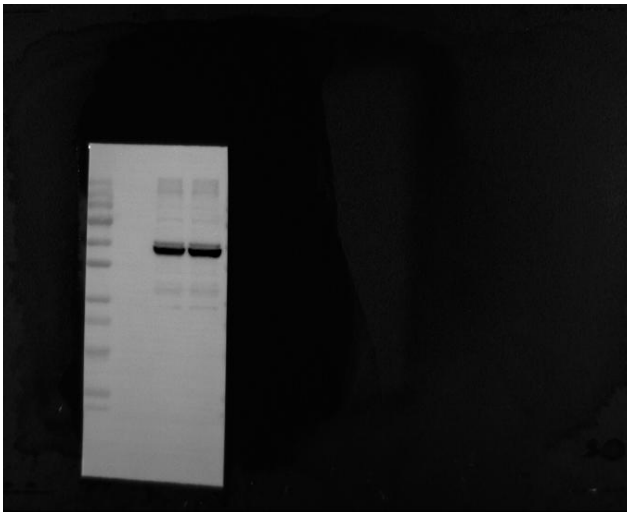

KDM8

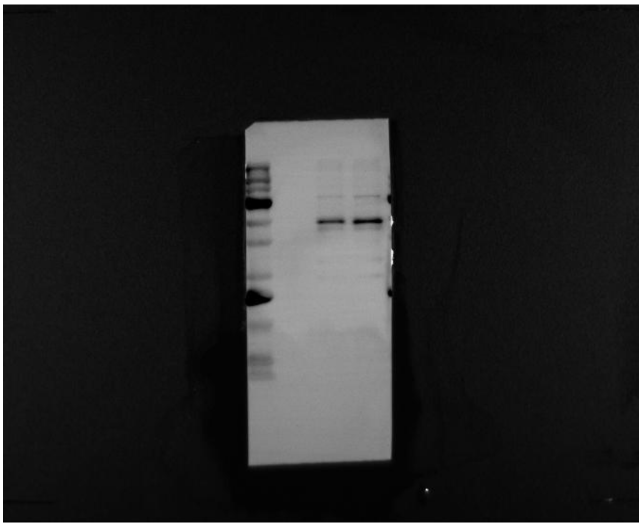

anti-IgG

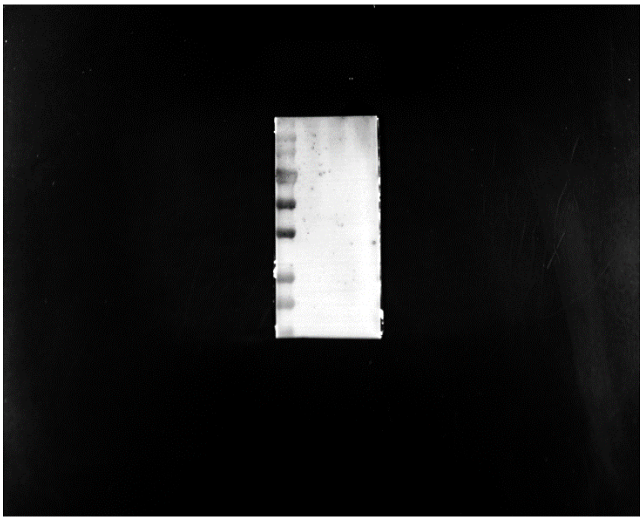

IP: HA

anti-HA

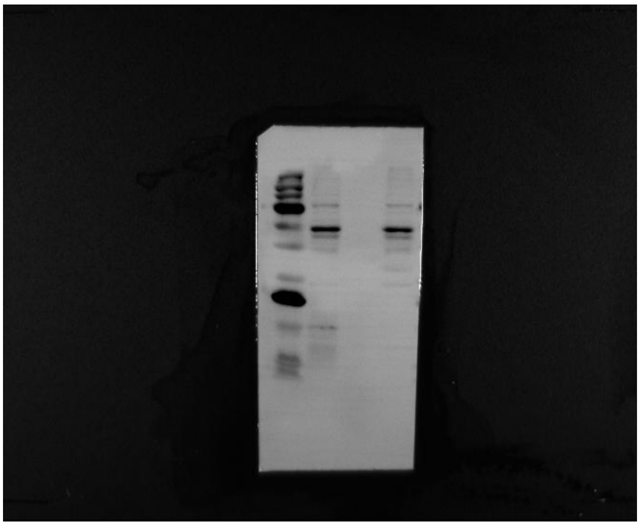

c-Myc

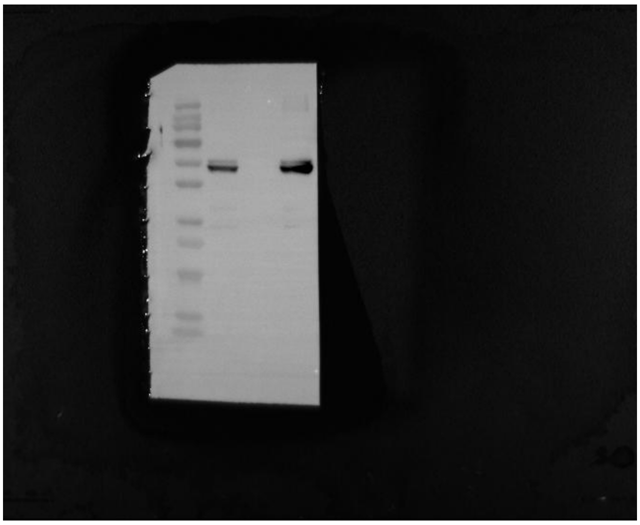

anti-IgG

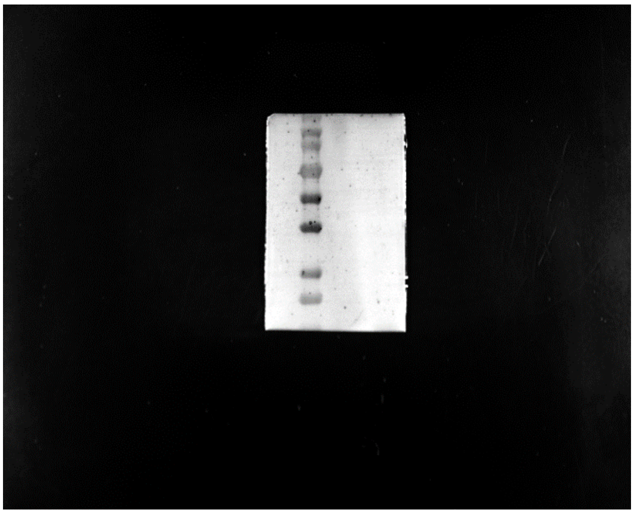

Figure 2D

Grouping from left to right:

SKOV3 (OE-Control, OE-KDM8, OE-KDM8+OE-cMyc, OE-KDM8+siRNA NC, OE-KDM8+siRNA c-Myc);

OVCAR3 (OE-Control, OE-KDM8, OE-KDM8+OE-cMyc, OE-KDM8+siRNA NC, OE-KDM8+siRNA c-Myc)

KDM8

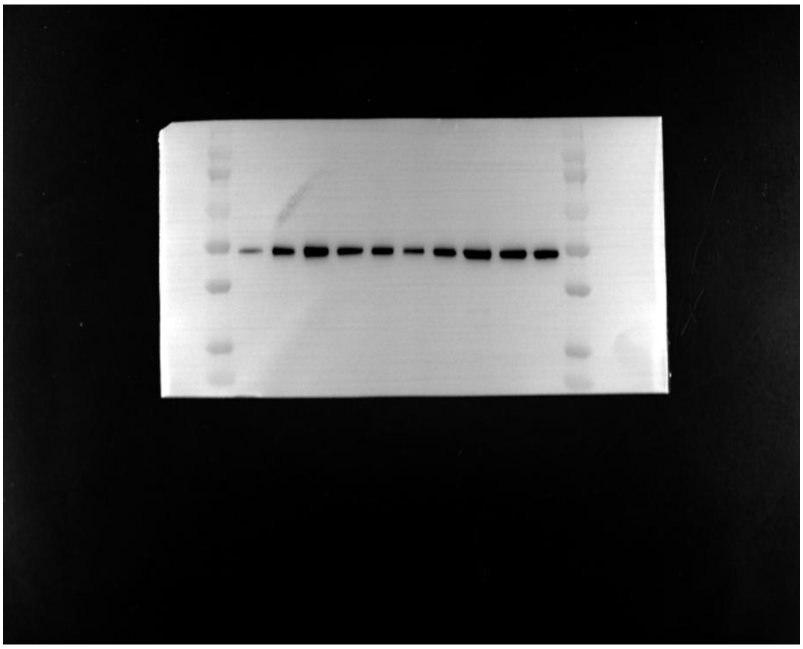

c-Myc

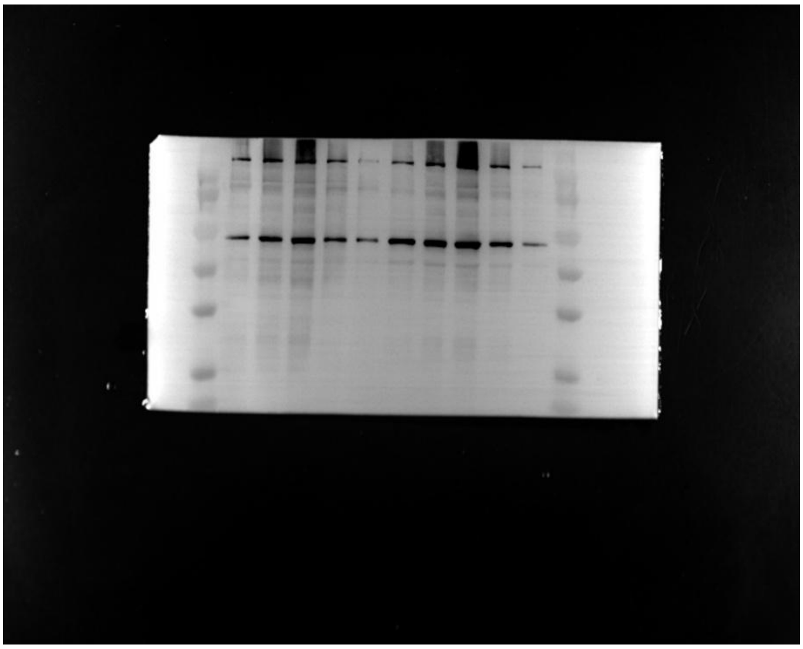

GAPDH

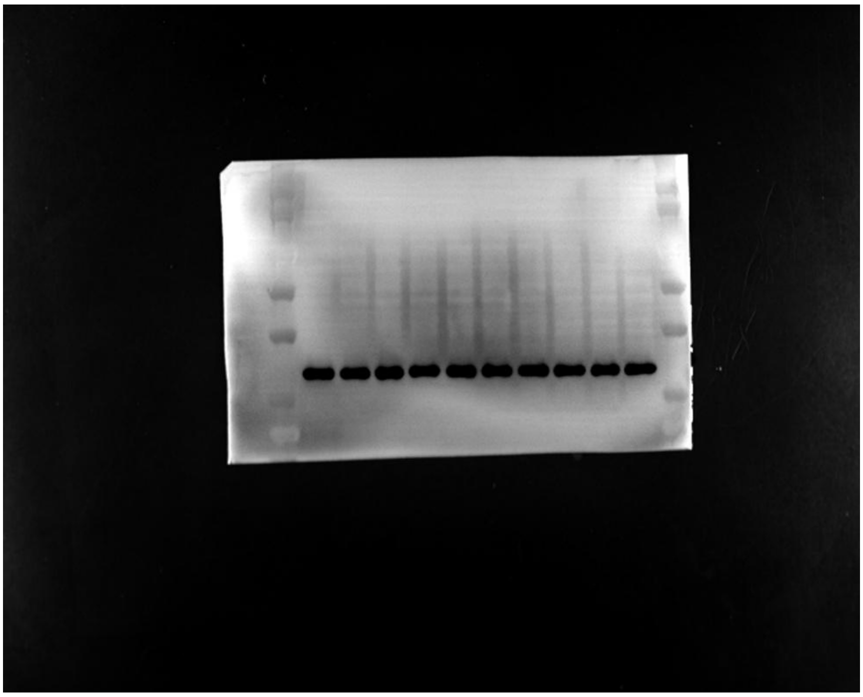

Figure 6A

Grouping from left to right: Control, OE-KDM8, sh-c-Myc, OE-KDM8+sh-c-Myc

KDM8

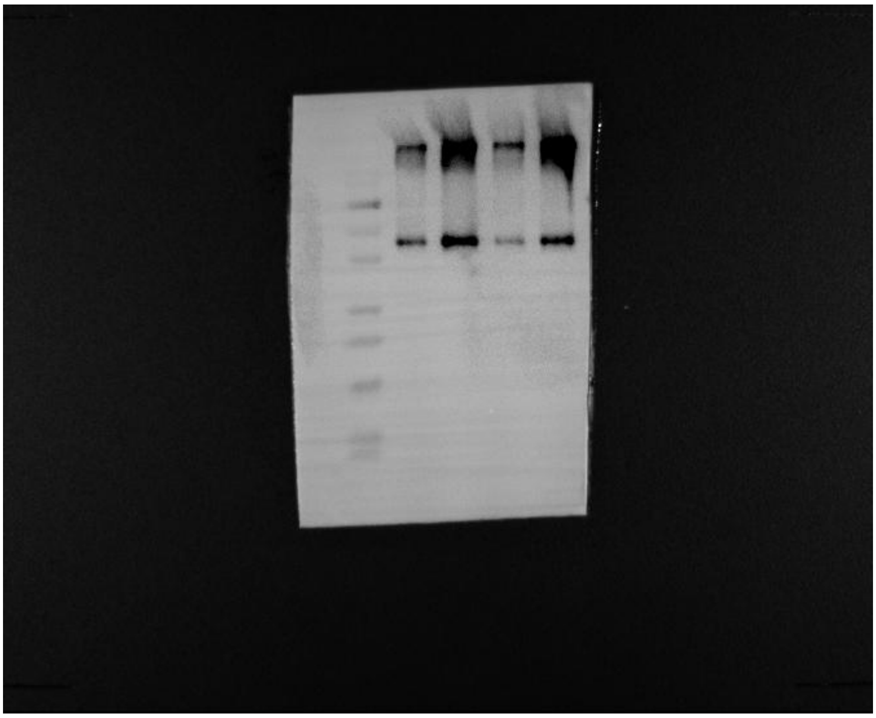

c-Myc

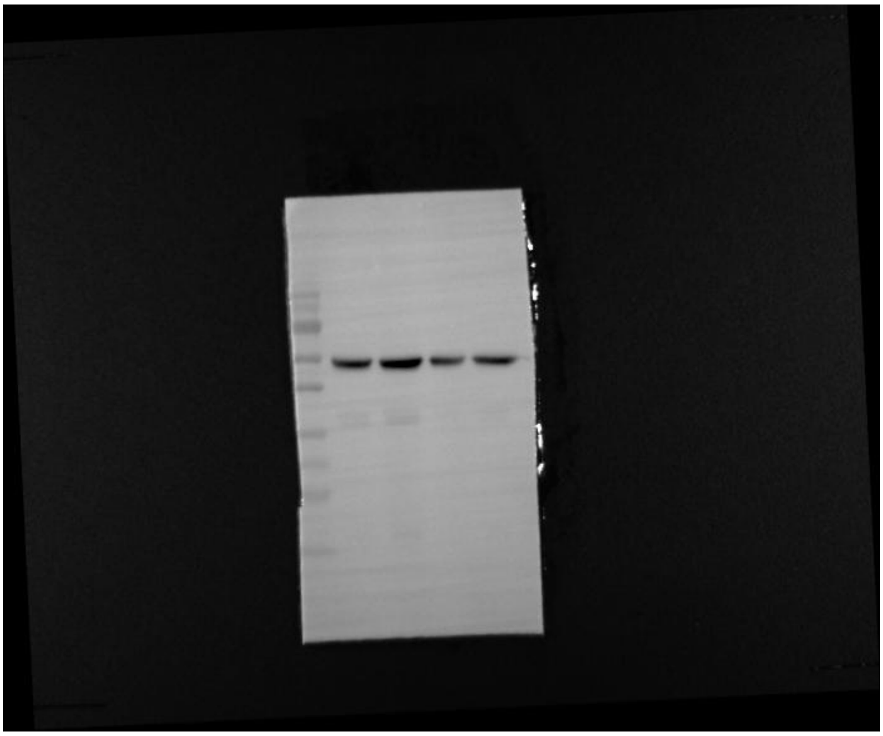

GAPDH

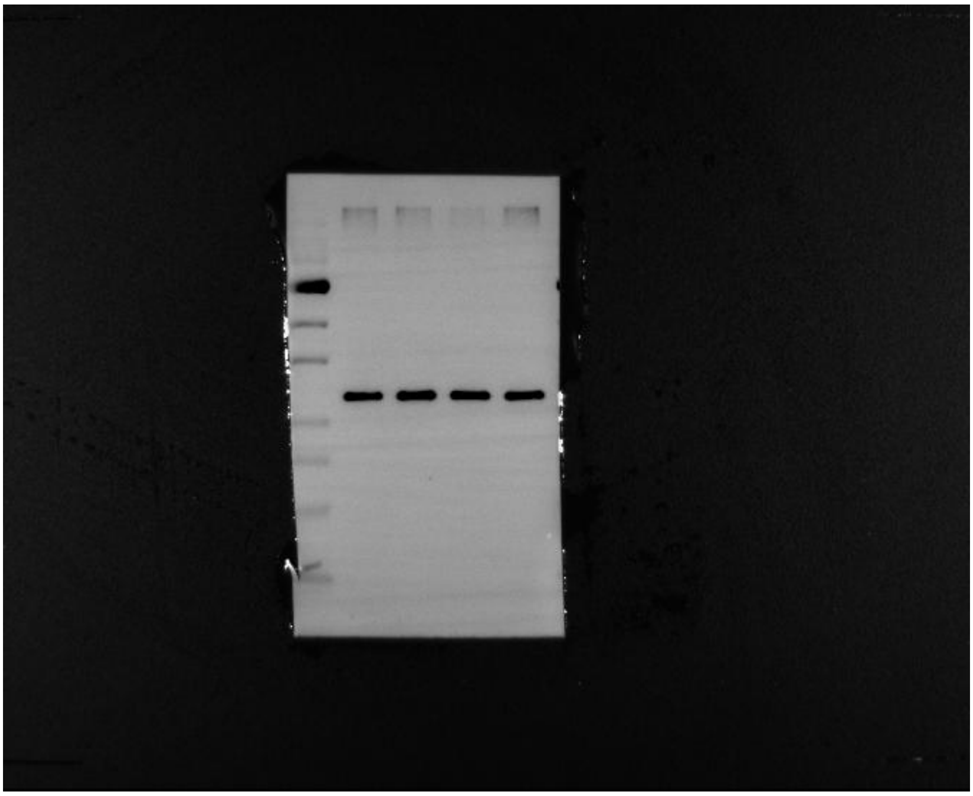

Supplement: Supplementary file 1 — Supplementary Information. [file 41598_2026_47344_MOESM1_ESM.pdf]
